# Asymmetric Cell Kinetics Genes: The Key to Expansion of Adult Stem Cells in Culture

**DOI:** 10.1100/tsw.2002.869

**Published:** 2002-07-09

**Authors:** James L. Sherley

**Affiliations:** ^1^ Biological Engineering Division Biotechnology Process Eng. Center Massachusetts Inst. of Technology, Cambridge, MA, USA; ^2^ Biological Engineering Division Massachusetts Inst. of Technology Building 16, Cambridge, MA 02139, USA

**Keywords:** stem cell, asymmetric cell kinetics, p53, tumor suppressor, IMPDH (inosine- 5’-monophosphate dehydrogenase), p^21waf1/cip1/sdi1^, cyclin-dependent kinase inhibitor, p63, Pten, transdifferentiation, transpotency

## Abstract

A singular challenge in stem cell research today is the expansion and propagation of functional adult stem cells. Unlike embryonic stem cells, which are immortal in culture, adult stem cells are notorious for the difficulty encountered when attempts are made to expand them in culture. One overlooked reason for this difficulty may be the inherent asymmetric cell kinetics of stem cells in postnatal somatic tissues. Senescence is the expected fate of a culture whose growth depends on adult stem cells that divide with asymmetric cell kinetics. Therefore, the bioengineering of strategies to expand adult stem cells in culture requires knowledge of cellular mechanisms that control asymmetric cell kinetics. The properties of several genes recently implicated to function in a cellular pathway(s) that regulates asymmetric cell kinetics are discussed. Understanding the function of these genes in asymmetric cell kinetics mechanisms may be the key that unlocks the adult stem cell expansion problem.

## OVERVIEW

The purpose of this article is to present a critical review of genes involved in the regulation of asymmetric stem cell kinetics. Though an essential process for normal tissue function in mammals, asymmetric cell kinetics are often overlooked in stem cell studies; and when they are considered, it is often with ambivalence, uncertainty, or confusion. An important aim of this review is to clarify the role of asymmetric cell kinetics in mammalian stem cell function, in particular as it relates to adult stem cells in somatic tissues. If asymmetric cell kinetics are an intrinsic property of adult stem cells *in vivo*, it can be anticipated that their study will significantly impact biomedical research efforts to isolate and expand adult stem cells for use in tissue engineering, gene therapy, and cell replacement therapy. Here, this concept will be explored from the perspective of how knowledge of asymmetric cell kinetics genes can better inform current research to propagate adult stem cells in culture for therapeutic purposes. Before beginning the main treatment of asymmetric cell kinetics genes, it will be instructive to review briefly several motivations that currently drive many stem cell research initiatives.

## ADULT STEM CELLS: STILL IN THE CELL THERAPY GAME

Presently, there is a tremendous revival of the concept of mammalian cell “de-differentiation” or “transdifferentiation” under way in the field of stem cell biology. The term transdifferentiation was originally coined by developmental biologists to describe the ability of apparently fully differentiated embryonic cells of one tissue type to become cells of a different tissue type in response to either surgical removal of an adjoining tissue segment or *in vitro* cell culture[1,2]. Transdifferentiation is now commonly used to describe a somewhat similar behavior attributed to stem cells in postnatal somatic tissues (i.e., adult stem cells). Several *in vivo* and *in vitro* studies suggest that adult stem cells originated in one tissue are able to produce cells with gene expression properties characteristic of cells in other tissues when they are transferred to a different tissue environment[3,4,5]. In particular, the ability of bone marrow–derived cells to yield cells with properties specific for liver hepatocytes[6,7,8], neurons[9,10], and cardiac myocytes[11] has received considerable attention, because of the relative ease with which bone marrow can be harvested from donors for clinical purposes[12,13].

Despite these remarkable developments, adult stem cell research has in no way captured the public interest in the way that embryonic stem cell research has. This is true even though embryonic stem cell research is besieged by highly emotionally charged political, moral, and ethical debates and is very likely to remain so for many years to come[13,14,15,16,17,18]. There are two factors that account for this surprising situation. The first of these is that the known tumorigenic properties of embryonic stem cells in postnatal tissues have been largely overlooked by both scientists and journalists. Remarkably, the basis for proving that human embryonic stem cells are totipotent is the demonstration that they form teratomas in postnatal tissues. Tumor tissues that originate from all three germ layers of the developing embryo (endoderm, mesoderm, ectoderm) are found in embryonic stem cell–derived teratomas[19]. Although this has received surprisingly little attention, the tumor-forming property of embryonic stem cells poses a perplexing barrier to their use for cell therapy. It is reasonable to expect that this barrier to therapeutic application will be no less formidable than the cancer problem itself.

The second factor contributing to the paradox in public attention to the two types of stem cells is the well-acknowledged challenge of expanding adult stem cells in culture[13,20,21]. Embryonic stem cells can divide exponentially in culture for long periods while maintaining their pluripotency. This ability is the basis for expectations that they can be produced in sufficient number for cell therapy applications. Of course, as noted earlier, the same properties that allow large-scale expansion of embryonic stem cells will also promote tumor formation in cell transplant recipients. Unlike embryonic stem cells, so far, adult stem cells have proven refractory to traditional methods of cell culture. As of this writing, the author is aware of only one report of long-term culture of putative adult stem cells with retention of their ability to produce differentiating progeny cells. Even in this report the exact origin and nature of the expanded cells were uncertain; and no studies were reported that address whether the cells have sustained transforming mutations[22].

Many view the difficulty of expanding adult stem cells in culture as a major barrier to their development for use in human cell replacement therapy[13,21,23]. Attempts to expand adult stem cells in culture are further hindered by the absence of molecular markers that can be used to identify and quantify adult stem cells. If the nature of the block to expansion of adult somatic stem cells in culture were fully understood, there might be more optimism for future success in adult stem cell research. Understanding *why* adult stem cell preparations senesce in culture is essential to efforts to bioengineer methods to promote their expansion in culture.

There are many biological mechanisms that may be essential for adult stem cell function *in vivo*, but whose disruption *ex vivo* constitutes the barrier to adult stem cell expansion. Included among these are growth factor signaling, extracellular matrix contact, telomere maintenance, cell-cell interaction, and intrinsic asymmetric cell kinetics. The last of these, asymmetric cell kinetics, is a defining property of adult stem cells. Recently, it was proposed that the asymmetric cell kinetics program of adult stem cells also poses a fundamental barrier to their propagation and expansion in culture[24,25]. The aim of this review is to consider this aspect of asymmetric stem cell kinetics with critical discussion of several genes recently implicated to function as determinants of adult stem cell kinetics.

Perhaps, it is not surprising that each of the five genes that will be considered for their potential role as asymmetric cell kinetics genes are already known to have functions in cell growth regulation. For two of the proteins encoded by these genes, the p53 tumor suppressor protein and inosine-5′-monophosphate dehydrogenase (IMPDH), the rate-limiting enzyme for guanine nucleotide biosynthesis, there is excellent evidence for their ability to regulate asymmetric cell kinetics. For the remaining three proteins, the cyclin-dependent kinase inhibitor $p21^{waf1/cip1/sdi1}$ and two other known tumor suppressors, p63 and Pten, the evidence is less clear. However, there are sufficient reports in the general area of stem cell kinetics to merit a consideration of whether these three proteins are also involved in the regulation of adult stem cell asymmetric cell kinetics. Each of these important cellular proteins will be discussed, in turn, from the perspective of studies that suggest a possible role in cellular mechanisms responsible for regulating the asymmetric cell kinetics of adult stem cells. Understanding how these proteins function to regulate asymmetric cell kinetics may yield keys to successful bioengineering strategies for expansion of adult stem cells in culture.

## ASYMMETRIC CELL KINETICS, THE ESSENCE OF ADULT STEM CELLS *IN VIVO*, THE BANE OF THEIR EXPANSION *EX VIVO*

The discovery of transdifferentiation by adult stem cells might have been predicted based on their unique differentiation properties. In postnatal somatic tissues, adult stem cells remain undifferentiated while simultaneously producing highly differentiated progeny cells. They accomplish this by asymmetric cell divisions that yield a new replacement stem cell and a transit cell daughter. Depending on the tissue type, transit cell daughters either differentiate directly or divide first to produce the variety of differentiated cell types that form the tissue[26,27]. Previously, there was one widely held view to account for the emergence of this pattern of cell differentiation in adult tissues. During embryonic development, cells destined to become adult stem cells were thought to undergo a restriction from pluripotency, the ability to give rise to all the different cell types in the body, to either multipotency or unipotency. Multipotent and unipotent stem cells are able to produce multiple cell types or only one cell type, respectively, but not all cell types. Now, the discovery of adult stem cell transdifferentiation may necessitate a revised explanation for the limited differentiation potential of adult stem cells in their tissue of residence[5,28,29].

Transdifferentiation may reflect a tissue mechanism in which local environmental factors dictate the differentiation of transit daughters and their descendants, while compartmentalized stem cells maintain their autonomous undifferentiated pluripotent state. Thus, the apparent tissue-specific restriction of stem cell potency that occurs during embryonic and fetal development may not reflect major changes in stem cell gene expression programs. Instead, the required gene reprogramming would take place in transit cells in response to local environmental signals from surrounding differentiated cells, extracellular matrices, and soluble factors. Recent studies support this alternative explanation[5]. Several groups have shown that when presumably ectoderm-derived adult neural stem cells are cultured alone or in contact with other cell types *in vitro*, they can produce progeny cells that express proteins and morphological features that are characteristic of myocytes, which are a mesoderm cell fate. Moreover, injection of genetically marked neural stem cells into mouse and chicken embryos indicated that these cells could yield progeny that develop into all three embryonic germ layers[5,30]. To reflect this different view of the cellular basis for adult stem cell transdifferentiation, this property is here defined as "transpotency". Transpotency is the adult stem cell property of latent multipotency or perhaps, more aptly, locally masked pluripotency (see Fig. 1).

Although asymmetric cell division is generally appreciated as an essential property of adult stem cells, the importance of their associated asymmetric cell kinetics is often overlooked. The ability of adult mammalian tissues to renew cells while maintaining a constant cell mass is owed to asymmetric stem cell kinetics[26,27,31]. The fate of all cells in transit daughter lineages is terminal division arrest. Only stem cells have division capacity that lasts for the entire mammalian life span[31,32]. For more than 3 decades, investigators have debated the exact mathematical form of asymmetric stem cell kinetics *in vivo*[27,29,32,33,34]. Some have argued that asymmetric kinetics are accomplished by pools of proliferating stem cells that divide to produce stochastically either new stem cells or differentiating progeny. Others maintain that it is accomplished by individual stem cells that produce one new undifferentiated stem cell and one differentiating progenitor cell at each division according to a determined program. In either case, the net result is a constant number of stem cells in adult tissues that produce differentiating progeny cells that ultimately all become nondividing, terminally arrested cells.

Rambhatla and others[25] recently proposed that a major barrier to the growth of adult stem cells in culture is their intrinsic asymmetric cell kinetics. *In vivo*, asymmetric cell kinetics provide tissue cell renewal in adult vertebrates that maintain near constant tissue cell mass. *Ex vivo*, the continuation of this cell kinetics program will result in increasing numbers of nondividing cells without a commensurate increase in stem cell number. As cultures are diluted for passage to new culture vessels, stem cells, which do not increase in number, are postulated to be diluted-out relative to a continually increasing fraction of nondividing cells. This basic cell kinetics eventuality has been proposed as the fundamental basis for replicative senescence of diploid mammalian cells in culture; and, thusly, the major barrier to the propagation and expansion of adult stem cells in culture[24,25; see Fig. 2]. Therefore, the key to solving the adult stem cell expansion problem may be found in understanding the nature of molecular mechanisms that regulate asymmetric cell kinetics.

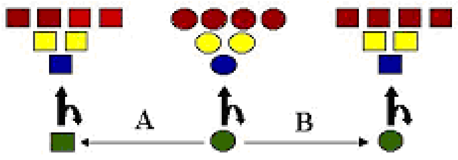

**FIGURE 1.** Transpotency, a manifestation of adult stem cell pluripotency**.** Shown are models for tissue turnover units produced by adult stem cells in somatic tissues[25,53]. In each turnover, a single adult stem cell (**green**) cycles with deterministic asymmetric cell kinetics to replace itself while producing differentiating transit cell progeny. (**A)** Transdifferentiation. Translocation of an adult stem cell to a different tissue environment may result in changes in the stem cell (**green circle** becomes **green square**) that lead to the production of transit cell progeny (**squares**) characteristic of the new tissue of residence. (**B)** Transpotency. An alternative mechanism to account for "transdifferentiation" is altered instruction of transit cells by the new tissue environment without any fundamental change in the potency of the stem cell. In this way, transpotency is synonymous with stable stem cell pluripotency associated with local instruction of transit cell differentiation.

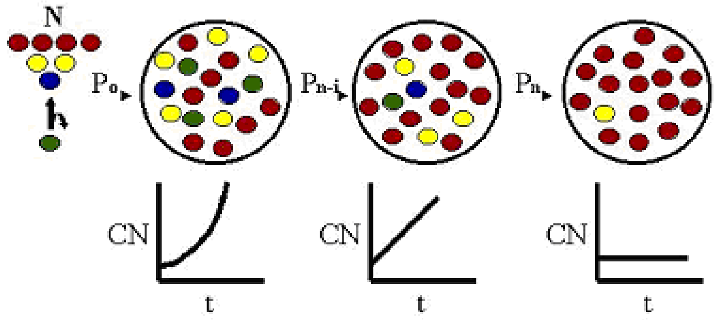

**FIGURE 2.** The asymmetric cell kinetics barrier to expansion of adult stem cells in culture. When tissue cells are explanted to culture, a large number (N) of adult stem cell–based tissue turnover units is obtained. **Green circles** represent adult stem cells, **blue** and **yellow circles** indicate differentiating transit cells, and **red circles** depict mature, terminally arrested cells that together comprise the basic turnover unit. It is postulated that in culture these tissue units regain their basic asymmetric cell kinetics, even though phenotypic differentiation of progeny cells is not restored. Because adult stem cell number does not increase during asymmetric cell kinetics, the stem cell fraction decreases with time as transit cells accumulate and fill up culture vessels. After the initial transfer of cells to culture (**Po**), successive dilutions of replete cultures (**Pn-i**) eventually yield a culture dilution in which no stem cells are transferred (**Pn**). This event is soon followed by a complete replicative arrest of the culture when all transit cells complete terminal maturation and division arrest[25]. Schematic graphs (cell number, **CN** vs. time, **t**) depict change in growth rate with successive passages in culture.

By discovering molecular pathways that allow reversible programming of adult stem cells so that they divide continuously with symmetric kinetics (i.e., exponential self-renewal), it may be possible to expand them in culture with retention of their innate ability to produce diverse types of differentiating transit cells given appropriate instruction.

## ASYMMETRIC CELL *KINETICS* GENES

Genes responsible for asymmetric cell kinetics went largely without discussion until the development of cultured cell systems that model deterministic asymmetric stem cell kinetics predicted to occur *in vivo*[25,35,36,37,38]. In contrast, many genes have been described that function in mechanisms for asymmetric cell *division*[39,40,41]. There are many examples of asymmetric cell divisions during embryonic development that result in phenotypically distinct daughter cells. Such asymmetric cell divisions are essential for normal embryonic development. As noted earlier, similar asymmetric divisions also occur during postnatal tissue cell differentiation in association with asymmetric stem cell kinetics. However, most investigations of asymmetric cell divisions reported to date have focused on developmental processes in embryonic tissues. Unlike postnatal asymmetric divisions, these embryonic asymmetric divisions are not associated with asymmetric cell kinetics. Therefore, the relationship of previously described genes involved in regulation of embryonic asymmetric divisions to adult asymmetric stem cell kinetics is uncertain[42].

Asymmetric cell *kinetics* is a special subset of asymmetric cell divisions. They occur primarily in late fetal and postnatal tissues and are characterized by production of one daughter cell that retains the capacity for division for the life span of the host and one daughter whose final descendants are all short-lived nondividing cells (i.e., a nondividing lineage). During the expansive phase of embryonic development, many phenotypically asymmetric cell divisions occur, but they are integrated with symmetric cell kinetics. Symmetric cell kinetics yield two lineages with equivalent division capacity. Although the lineages may be phenotypically distinct, they are equivalent for producing long-lived dividing progeny cells. The first evidence for the establishment of stem cells with asymmetric cell kinetics is in the fetus when adult tissue architecture begins to emerge[43,44]. Until late in embryonic development, asymmetric cell kinetics would be quite disruptive to embryonic tissues.

Genes involved in the regulation of asymmetric cell kinetics might be expressed in adult stem cells themselves or in cells that either interact with adult stem cells or produce substances that interact. The second and third mechanisms have been the focus of research to define the nature of stem cell niches. There is a large body of work, in invertebrate and mammalian species, describing genes whose expression in cells adjacent to stem cells may modulate stem cell division and differentiation. The reader is directed to excellent reviews on this topic[3,28,45]. It is probable that similar extrinsic mechanisms will contribute to the regulation of adult somatic stem cell asymmetric cell kinetics[28,45]. However, to date, the best candidates for genes that regulate mammalian adult somatic stem cell kinetics appear to function in a cell-autonomous fashion, as will be discussed here.

## p53

The first demonstration that mammalian cells could divide with determined asymmetric cell kinetics also identified the first candidate for a cell-autonomous asymmetric cell kinetics control gene. This was the p53 tumor suppressor gene in adult-derived murine mammary epithelium cells in culture[35,36]. When up-regulated by less than 50% above the basal level of endogenous wild-type p53 in these cells, p53 induced a switch from symmetric cell kinetics which produce two dividing daughters (underlying the exponential kinetics that typify established cell lines) to determined asymmetric cell kinetics. The asymmetric cell kinetics were characterized by divisions producing one continuously dividing daughter and one viable daughter arrested in G1/S of the cell cycle[35]. Subsequent studies with p53-null murine fetal fibroblasts demonstrated that simple restoration of wild-type p53 to basal expression levels restored determined asymmetric cell kinetics[25]. The same cell kinetics program was observed for cells in presenescent cultures of murine fetal fibroblasts and human lung fibroblasts[25,35]. These observations led to the proposal that p53-dependent asymmetric cell kinetics is a major barrier to the propagation of adult somatic tissue cells in culture[24,25]. Consistent with this hypothesis, a well-known requirement for immortalization of both murine and human cells in culture is loss of wildtype p53 function[46,47].

An important consideration is whether these *in vitro* findings with p53 are relevant to adult stem cell mechanisms *in vivo*. In several examined epithelial tissues, p53 protein expression is detected primarily in basal cells localized in regions thought to harbor adult stem cells[48,49]. Several different lines of transgenic p53 knockout mice have been derived. The consistent predominant phenotype of the p53 knockout mice is early development of diverse tumors[50,51,52]. This finding is consistent with well-established ideas that increased symmetric stem cell divisions induce elevated rates of carcinogenesis[31,53]. Previously, other hypotheses have been advanced to account for the remarkable cancer phenotype of these mice (e.g., DNA damage checkpoint arrest deficiency; [54]). However, consistent with the hypothesis of deficient asymmetric stem cell kinetics, in *ex vivo* studies, cells cultured from diverse tissues of p53 knockout animals no longer senesce in culture[51,55]. The absence of senescence by cultures of p53-null tissue cells is consistent with abrogation of the asymmetric cell kinetics barrier to adult stem cell expansion by removal of p53-dependent regulation[24,25]. Of course, the many genomic alterations that are associated with p53-deficient cells render them quite useless as expanded “stem cells”[54,56].

## IMPDH

Compared to *in vivo* experimental models, *in vitro* cultured cell models offer greater accessibility and experimental control for molecular and biochemical analyses. These advantages of cultured cell models were instrumental in the discovery of an essential component of the mechanism by which p53 expression modulates cell kinetics symmetry. Cultured cells engineered to have investigator-controlled expression of p53 were used to show that p53 expression causes down-regulation of IMPDH. Although the responsible molecular mechanism has not been determined, p53 expression is associated with coordinate down-regulation of IMPDH mRNA, protein, and cellular activity[57,58]. Two lines of evidence suggest that IMPDH down-regulation is required for p53-dependent asymmetric cell kinetics. First, nucleotide salvage compounds that promote the formation of the product of the IMPDH reaction (e.g., xanthosine monophosphate, XMP) prevent the p53-induced switch to asymmetric cell kinetics[35,57,58]. Second, forced expression of an IMPDH transgene prevents p53-dependent asymmetric cell kinetics[25,59].

The demonstration that IMPDH is a rate-determining component of the p53-dependent pathway for asymmetric cell kinetics invoked a role for cellular guanine nucleotides in asymmetric cell kinetics control mechanisms. Initial studies suggested that the critical guanine nucleotide(s) is a ribonucleotide(s)[35,57]. However, the exact identity of the acting guanine ribonucleotide(s) and its target(s) have not been determined.

In mice and humans, there are two reported IMPDH genes, designated type I and type II[60]. The human type II IMPDH gene shows elevated expression in malignancies and other highly proliferative growth states. In contrast, the type I gene does not exhibit growth-related regulation. The murine type II IMPDH gene mediates p53-dependent asymmetric cell kinetics in the cultured cell models described earlier[25,58,59]. It is noteworthy that the type II IMPDH gene is a member of a recently defined set of genes whose expression is associated with hematopoetic stem cell-enriched populations[61]. Transgenic knockout of the type II IMPDH genes in mice has not yielded additional information on the possible role of IMPDH in adult stem cell kinetics. IMPDH II gene knockout resulted in embryonic lethality, even though the type I gene appeared to be functional[62].

## p21$^{waf1/cip1/sdi1}$

The p21$^{waf1/cip1/sdi1}$ (p21) cyclin-dependent kinase inhibitor is much better known than IMPDH as a p53-responsive gene target. From the time of its identification, p21 has been considered a key mediator of p53-dependent checkpoint arrest in response to DNA damaging agents[38,63]. However, two recent reports suggest a possible role for p21 in homeostatic controls for adult stem cell kinetics. Because this emerging view of p21 cellular function is largely based on studies in p21 knockout mice, the exact nature of the involvement of p21 in stem cell function is less clear than that of p53 and IMPDH. Topley and others[64] studied alterations in keratinocyte cell kinetics in the skin of p21 knockout mice. Two properties of cells isolated from the epidermis of these mice indicated an expanded pool of either keratinocyte stem cells or primitive epidermal progenitor cells. *In vitro* culture analyses showed that p21 knockout mice had high numbers of clonogenic keratinocytes that occurred at frequency of about 1 in 1,000 isolated primary keratinocytes. In contrast, no similar clonogenic cells were detected in primary keratinocyte cells derived from wildtype mice. Compared to cells from wildtype mice, keratinocytes derived from p21 knockout mice and, subsequently, retrovirally marked, had a 20-fold greater capacity for what appeared to be clonal repopulation of entire hair follicles. This second property supports the authors' conclusion that p21 may function cell-autonomously to regulate the proliferation and/or differentiation potential of either adult keratinocyte stem cells or very primitive keratinocyte progenitors for epidermis and hair follicles.

Cheng and others[65] evaluated effects of p21 gene knockout on hematopoietic stem cell (HSC) kinetics. In serial transplantation assays, bone marrow cells from p21 knockout mice showed a greatly reduced ability to reconstitute the hematopoietic system of lethally irradiated recipient mice. Because this phenotype was demonstrable by transfer of mutant cells into wildtype recipient mice, p21 appeared to act in a cell-autonomous fashion. Flow cytometry analysis indicated that the HSC compartment of knockout mice had a significantly reduced fraction of noncycling cells. The authors proposed that p21 deficiency compromised the ability of HSCs to maintain a quiescent state that serves to protect them from toxic injury and that is required for hematopoiesis homeostasis.

Consistent with the proposal that p21 is an important determinant of adult stem cell kinetics, p21 knockout mice exhibit an early cancer phenotype (though with a longer latency than p53 knockout mice), and, in fact, 65% of the detected tumors are hematopoietic in origin[66]. Like p53 deficiency, p21 deficiency also promotes an extended life span for cultured human fibroblasts[67,68], and p21 expression is elevated in senescent cell cultures[67]. It is noteworthy that, although p21 is still widely held to be a mediator of p53-dependent checkpoint arrest in response to DNA damage, p21 knockout mice failed to give the full checkpoint deficiency phenotype noted for p53 knockout mice[69].

Although p21 is viewed as a universal cyclin kinase inhibitor, capable of inhibiting several different G1 and S phase-specific cyclin kinase complexes[70], surprisingly, in response to its p53-induced expression, only the cyclin-dependent kinase 2/cyclin E complex (cdk2/E) shows detectable inhibition[71,72]. This S phase-promoting cyclin kinase complex has been implicated to regulate the activity of DNA replication machines, and, thus, is a plausible component of a p53-based pathway for asymmetric cell kinetics[73]. Whether cdk2/E is a downstream component of a p53-p21 pathway for regulation of adult stem cell kinetics will require more research targeted at interrogating this possibility.

## AN EMERGING GENETIC CONTROL PATHWAY FOR ADULT STEM CELL ASYMMETRIC CELL KINETICS

The described *in vitro* studies with cultured model cells make it clear that IMPDH down-regulation is a rate-determining factor for p53-induced asymmetric cell kinetics. However, a similar dependency between p53 and p21 has not been directly established to date. In addition, the findings with p21 knockout mice do not necessarily indicate a function in the regulation of asymmetric cell kinetics. Cheng and others cautiously point out that effects on other processes such as stem cell quiescence, cell differentiation, and apoptosis could also be responsible for their observations[65]. In fact, given current ideas on the location of stem cells in epithelial tissues, the microanatomical distribution of p21 expression in epithelial tissues does not fit well with a function in stem cells. P21 protein does not show significant expression in basal regions presumed to harbor stem cells based on differentiation and cell kinetics mapping experiments. Instead, in both murine and human epithelia, p21 is highly expressed in regions of newly produced terminally differentiating cells[74].

Allowing that the phenotype of p21 mice does reflect some degree of involvement in asymmetric stem cell kinetics, then adult stem cell kinetics control may be considered to result from p53-dependent “counter-regulation” of IMPDH and p21, IMPDH activity being down-regulated in concert with up-regulation of p21 (see Fig. 3). In this model, neither p53-responsive target would be sufficient to maintain asymmetric cell kinetics alone. An important question for future investigations is how might IMPDH function and p21 function be integrated to produce daughter cells with different cell kinetics fates.

Both p21 and IMPDH receive regulation inputs from extracellular and intracellular factors other than p53, and thus their link to p53 may be only one of several inputs for maintenance of stem cell kinetics. Certainly, asymmetric inheritance of any of these three gene products by one of the daughter cells might be an important aspect of the control mechanism. At first, this may seem implausible for an essential enzyme like IMPDH. However, the existence of two isoenzymes in mammalian cells opens up the possibility of asymmetric inheritance of one isoenzyme for the purpose of driving asymmetric cell kinetics.

## p63

It is improbable that p53, IMPDH, and p21 are the sole regulators of asymmetric cell kinetics. In addition to the possibility of other genes that, like p53, regulate the actions of IMPDH and p21,

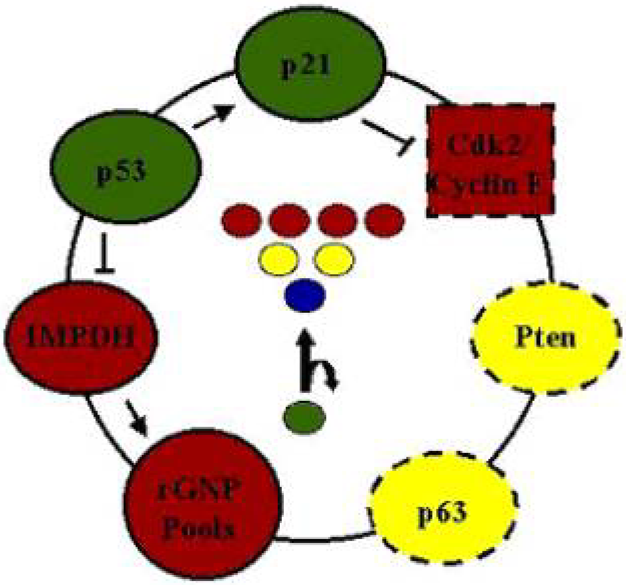

**FIGURE 3.** Emerging asymmetric cell kinetics genes**.** Diagrammed are genes implicated in the regulation of asymmetric cell kinetics. For components drawn with **solid lines**, there is good evidence that their function is rate-determining for asymmetric cell kinetics. Weaker evidence is available for the involvement of components drawn with **dotted lines**. Expression of **green** components promotes asymmetric cell kinetics; whereas **red** components promote symmetric kinetics that may support the exponential expansion of adult stem cells in culture. The nature of possible effects of components in **yellow** is unclear. P53 may be a central component of a cellular pathway that regulates the cell kinetics of adult stem cells. It simultaneously down-regulates IMPDH expression while up-regulating p21 expression, two events that favor the maintenance of asymmetric cell kinetics.

there are likely to be many cellular components whose response to changes in IMPDH and p21 are required for asymmetric stem cell kinetics. Given the implication of small molecules like guanine ribonucleotides, the target range of p21, and the cellular endpoints of active cycling and S phase arrest, the existence of additional downstream asymmetric cell kinetics control genes seems certain.

In keeping with this expectation of more asymmetric stem cell kinetics genes, recently, two additional genes have been implicated to play a role in regulating adult stem cell kinetics. The first, p63, is itself a member of the p53 gene family[75, reviewed in 76]. Different transcription units for p63 produce variant proteins that can either transcriptionally activate known p53-responsive genes (including p21) or interfere with p53-dependent gene transcription[75,76]. Like p53, p63 protein is expressed in basal cells in murine and human epithelia. Unlike p53, p63 is not expressed in rare cells, but instead is frequently detected in basal cells in diverse epithelial tissues[75,76,77,78]. This finding indicates that p63 expression is not restricted to epithelial stem cells that are estimated to contribute less than 1 in 1,000 cells in epithelial tissues[26,27,31].

Despite its pattern of high frequency of cellular expression, Yang and others have proposed that p63 might be a determinant of stem cell kinetics because of the epithelial phenotype of p63 knock-out mice[76,79]. These animals die as newborns with many poorly developed or undeveloped epithelial tissues[79,80]. Because vestigial cells that express mature differentiation markers in otherwise degenerate tissues could be found in late stage embryos (“nonregenerative differentiation”), Yang and others proposed that the animals had a defect in asymmetric stem cell division[76,79]. This view must be taken cautiously because of the nature of the p63 knockout phenotype, which is largely embryonic in origin. Both the report by Yang[79] and a simultaneous report by Mills and others[80] showed that developmental defects in p63 knockout embryos are noticeable as early as embryonic day 9.5 (the earliest time that p63 can be detected in the embryo; [80]) and 10.5. These stages are prior to the time that asymmetric somatic tissue stem cells are thought to be established[43,44].

Although the evidence, as discussed above, indicates a role for p63 in embryonic development, it does not rule out an additional role in adult stem cell function. In fact, a recent report describes a strong association between p63 expression and putative adult stem cells in the human corneal epithelium[78]. An excellent map of the *in vitro* proliferative capacity of human corneal epithelium has been constructed. The basal layer of the corneal limbus, which is thought to contain stem cells that cycle slowly *in vivo*, has a higher fraction of cells with the greatest *in vitro* proliferative capacity (called holoclones) than other regions of the epithelium[81]. Immunohistochemical analysis showed that p63 was frequently expressed in basal cells of the limbus. Holoclones derived from corneal cell cultures (15% of total limbus-derived cell clones) expressed significantly higher levels of p63 protein as compared to cell clones with diminished proliferative capacity and presumed to originate from early and late transit cells. Similar relationships between proliferative capacity and p63 expression were noted in analyses with human epidermis that yield similar, but somewhat rarer (6.6%) holoclones that are thought to derive from either epidermal stem cells or earlier progenitors[81].

Whereas the association between p63 expression and epithelial holoclones is certainly provocative, there is still sufficient cause for caution in assigning p63 a direct role in adult stem cell function. The fact that p63 expression is so frequent in basal epithelial cells and that holoclones are so frequent in epithelial cultures raises an important concern. These data do not fit well with the concept that stem cells are rare in adult tissues, where rare is defined as existing at a frequency that is <1/1,000. Therefore, either ideas on the frequency of stem cells in some epithelial tissues must be revised, or the conclusions regarding p63 and holoclones must be revised to consider, respectively, functions in cell types and origin from cell types other than adult somatic stem cells.

## Pten

The second new candidate for involvement in regulation of adult stem cell kinetics, Pten, is also a known tumor suppressor protein. The Pten gene encodes a phosphatase that is frequently mutated in a variety of human cancers; and germline mutations in Pten predispose for an overlapping spectrum of diseases that include cancer syndromes and developmental defects[82,83]. Again, studies with gene knockout mice are the basis for considering Pten as a regulator of adult stem cell kinetics. Traditional knockout of Pten results in embryonic lethality, presumably because of a role for the protein in early embryonic development. Groszer and others used a conditional, targeted Cre-loxp approach to develop mice that underwent Pten knockout primarily in CNS cells at mid-gestation[84]. These mice exhibited brain abnormalities associated with increased neuronal proliferation and decreased apoptosis. *In vitro* studies with neurosphere cultures developed with cells from knockout mice indicated that Pten deficiency increased neural stem/progenitor cell proliferation. Moreover, the effects of the mutation were specific for neural stem cell kinetics, as alterations in the differentiation of progenitor cells were not observed. Though these effects were demonstrated with cultured cells, ongoing production of differentiated cells in neurosphere cultures precluded the determination of whether Pten effects on stem/progenitor proliferation were cell-autonomous[84].

Like p63 knockout animals, Pten Cre-loxp knockout mice die immediately after birth, and the mutant phenotype is for the most part embryonic in origin. Unlike Yang and others in their work on p63[79], Groszer’s group does not propose that Pten is an effector of adult stem cell kinetics. As noted earlier, the distinction between effects in embryonic stem cells and adult stem cells is an important one to make, though it is often confused. Unlike p53, Pten deficiency leads to embryonic lethality. The embryonic requirement for Pten function may continue into midgestation and even the neonatal phase of brain development. Thus, the mutant phenotypes reported by Groszer and others for Pten knockout in the CNS[84] are most probably due to Pten function in embryonic stem cell kinetics, not adult.

For both Pten and p63, future success in defining the nature of their involvement in adult stem cell kinetics *in vivo* will require experiments that avoid the embryonic lethality and pathology, respectively, encountered in transgenic knockout studies. This might be accomplished by one of two related approaches. Production of transgenic mice derived with inducible p63 or Pten genes would allow an evaluation of the effects of their overexpression on cell kinetics during fetal, neonatal, and adult tissue development. Similarly, *conditional* transgenic knockout mice would allow investigation of their requirement for tissue stem cell kinetics at prescribed stages of development. The development of such transgenic mice is well within the capability of modern molecular genetics technology.

## MECHANISMS OF CELL-AUTONOMOUS ASYMMETRIC INHERITANCE

A defining feature of asymmetric cell divisions that characterize cell differentiation during embryonic development is asymmetric inheritance. Asymmetrically inherited factors can be intracellular or extracellular. Differentiating cells have been shown to asymmetrically partition existing proteins or RNAs and to exhibit unequal expression of specific genes. These mechanisms result in daughter cells with significantly different amounts of critical cell fate determinants. Daughters can also “inherit” different extracellular environments (e.g., basal vs. apical location, specific cell-cell interactions) that may either independently induce cell fate decisions or promote asymmetric localization of intracellular fate determinants prior to cell division[39,40,41,85].

Adult stem cells *in vivo* might also employ asymmetric inheritance mechanisms to achieve asymmetric cell kinetics. There is evidence that the adult stem cell niche in both invertebrates and vertebrates plays a role in regulating adult stem cell kinetics[28,45]. However, as discussed, the demonstration of asymmetric cell kinetics by isolated cells *in vitro*[25,35,36,86] suggests the existence of cell-autonomous asymmetric inheritance of determinants of asymmetric cell kinetics. The five genes considered in this article have not been specifically evaluated for expression symmetry between cycling and noncycling daughters. Of course, finding that one or more of them were expressed asymmetrically might lead to an understanding of the molecular basis of deterministic asymmetric cell kinetics. However, such observations would raise a new question, which is, in fact, the fundamental issue. What types of cellular mechanisms could account for cell-autonomous asymmetric inheritance?

The simple explanation that asymmetric cell kinetics could be based on the stochastic partitioning of “cell kinetics determinant” molecules between two daughter cells is deceptive. The basic idea is that for a freely diffusing determinant, it is unlikely that daughter cells will inherit exactly the same number of molecules. The cell inheriting the larger (or smaller) share, however so slight, would be fated, for example, to become the continuous cycling stem cell. However, it is difficult to envision a biological control mechanism that would be so finely tuned as to function robustly with this basis for operation. Synthesis and turnover of the cell kinetics determinant would have to be exquisitely well controlled to prevent failed kinetics programs due to stochastic fluctuations in factor concentration.

At first, a cell kinetics determinant that exists in a single copy, so that only one daughter can inherit it, seems like an elegant solution to the problems of stochastic variation in the concentration of an asymmetrically inherited determinant. For example, a single-copy messenger RNA might seem an effective mechanism. However, such a cell kinetics determinant would, in fact, have probabilistic properties that would result in its failure to support the deterministic asymmetric cell kinetics. Two different molecular mechanisms might yield a single-copy mRNA. A gene might be of such extreme length or of such poor transcriptional efficiency that, on average, only one complete gene transcript was produced before each stem cell division. However, either of these mechanisms might occasionally yield more than 1 or no complete copies of the mRNA in daughter cells. Such events would be catastrophic for tissue units *in vivo*. In addition, as in the case of asymmetric inheritance based on unequal determinant concentration, strict coordination of the synthesis and turnover of a single-copy determinant with cell division rates would also be essential for reliable function.

The single-copy determinant model for cell-autonomous asymmetric inheritance becomes more plausible, if two additional features are invoked. First, the cell kinetics determinant must be stable on the time scale of mammalian life spans. Second, production of new copies of the cell kinetics determinant must be tightly restricted. Consider a stem cell bearing a stable single-copy determinant that confers stem cell kinetics. At division only one of the daughter cells inherits the determinant, and it becomes the new stem cell. A general inhibitor is required in *both* daughter cells to prevent the production of new stem cell kinetics determinants. Without the determinant, the other daughter would be prevented from acquiring a stem cell fate, thereby rendering it permissive to differentiation instruction.

This discussion would not be complete without consideration that the actual answer to the fundamental question of cell-autonomous asymmetric inheritance may be that determined asymmetric cell kinetics *in vitro* only *appear* to be cell-autonomous. Specific communication between newly divided daughter cells could be the key to the mechanism. For example, both new daughters may "compete" to instruct each other to be the stem cell daughter with only one winner possible. The loser would function as a stem cell, whereas the winner would initiate a transit cell differentiation program. Instruction might be in the form of inhibitory cell-cell contact or a mutually secreted factor that inhibits/promotes the acquisition of stem cell kinetics. A mechanism of this type has been suggested for apparently cell-autonomous binary fate decisions during development in *C. elegans*[85]. Fairly simplistic interacting regulatory loops could result in a faithful mechanism to insure asymmetric cell kinetics by the stem cell daughter. The schemes considered here provide a general framework for future investigations of asymmetric cell kinetics control genes. One or more of them may be involved in the elaboration and regulation of key cell kinetics determinants that exhibit cell-autonomous asymmetric inheritance or that form stem cell-transit cell regulatory loops that yield asymmetric daughter cell kinetics fates.

## A GENETIC BASIS FOR BIOENGINEERING THE EXPANSION OF ADULT STEM CELLS IN CULTURE

To date, no rationale approach to the general problem of expanding adult stem cells in culture has been described. The asymmetric cell kinetics genes described in this review (summarized in Fig. 3) have potential to become the basis for the development of such an approach. Present research is focused on the identification of specific growth factors or cellular proteins that might promote stem cell propagation in culture. Factors that increase adult stem cell viability, proliferation, and replicative life span or that prevent cell differentiation are common targets for research in this area. These investigations are essential to the development of a complete understanding of stem cell function, but it is unlikely that they will lead to a solution to the expansion problem; unless they also attend to the barrier posed by intrinsic asymmetric cell kinetics.

P53 and IMPDH have emerged as rate-determining factors in a cellular pathway that regulates asymmetric cell kinetics; and p21 and p63 have some characteristics that implicate them as components of the same or related pathways for cell kinetics control. Continued investigation of the role of these genes in adult stem cell kinetics will be required to elucidate the complete genetic structure of adult stem cell kinetics control mechanisms. Development of a detailed understanding of molecular and biochemical features of this pathway is the first step in bioengineering methods to expand adult stem cells in culture. A method to reversibly convert asymmetric cell kinetics to symmetric cell kinetics is the critical requirement for success. Asymmetric cell kinetics genes may hold the secrets that must be revealed to realize the goals of identifying adult stem cells and procuring them in the quantities required for successful therapeutic development.

## ACKNOWLEDGEMENTS

Many thanks to J. Lansita, K. Panchalingham, R. Helmi, and Drs. J.-F. Paré and C. Semino for critical review of the manuscript.
